# Data of temperature and relative humidity in a historic library in Portugal

**DOI:** 10.1016/j.dib.2019.103788

**Published:** 2019-03-15

**Authors:** Eva Schito, Luisa Dias Pereira, Daniele Testi, Manuel Gameiro da Silva

**Affiliations:** a Department of Energy, Systems, Territory and Constructions Engineering (DESTEC), University of Pisa, Pisa, Italy; b ADAI, LAETA - Department of Mechanical Engineering, Faculty of Sciences and Technology, University of Coimbra, Coimbra, Portugal

## Abstract

The correct preservation of old and rare books in libraries needs suitable values of temperature and relative humidity. This paper shows the hygrothermal data acquired in a historic library, the Baroque Library, of the University of Coimbra, Portugal, where several old and rare books are stored. The data were acquired during a 6-month monitoring campaign. In particular, in this paper the spatial and temporal variations of these two parameters are analysed. The data presented in the article are related to the research article entitled “A procedure for identifying chemical and biological risks for books in historic libraries based on microclimate analysis” [1]*.*

**Table:** Specifications table

Subject area	*Engineering*
More specific subject area	*Building Physics*
Type of data	*Graphs and carpet plots*
How data was acquired	*Monitoring campaign through temperature and relative humidity sensors (*HOBO UX100-003 data loggers*)*
Data format	*Raw, analysed*
Experimental factors	*No pre-treatment*
Experimental features	*The data are analysed to verify the presence of spatial or temporal span in a historic library in Portugal, where old and rare books are stored.*
Data source location	*Baroque Library of University of Coimbra, Portugal*
Data accessibility	*Data are within this article*
Related research article	*“A procedure for identifying chemical and biological risks for books in historic libraries based on microclimate analysis”*[1]*.*

**Table:** 

**Value of the data** • The data in this paper can be useful for researchers, providing real indoor environmental parameters of historic libraries. • The data can help researchers in the identification of retrofit strategies in similar cultural contexts. • The data can be useful for comparison with other hygrothermal data, acquired in other historic libraries. • The data can be useful for further research in the same library, evaluating the variation of thermal indoor environment in different conditions of external climate and visitors' presence.

## 1 Data

The data shown in this article are related to the research article “*A procedure for identifying chemical and biological risks for books in historic libraries based on microclimate analysis”* [1]*.* They show some additional analyses of indoor microclimate in the Baroque Library of the University of Coimbra, Portugal. In the library, temperature and relative humidity were gathered during a 6-month monitoring campaign in several points of the rooms, to verify if the internal microclimate would lead to damage and deterioration of the 40 thousands old and rare books that are hosted inside [2], [3]. The Library has a total volume of 7,000 m^3^ and an area of about 1,250 m^2^ distributed along three floors (the Noble floor, the Intermediate Floor, and the Academic Prison). It is characterized by its unique richly ornamented oak-wood shelves, ceilings decorated with trompe-l'œil, arches and paintings. There are thick masonry walls (up to 2-m wide), a moderate glazed area and no HVAC system. The internal conditions of the Library have been altered due to tourist flows (in 2017, half-million people visited the Baroque Library): thus, it can be considered as a touristic attraction, having rare books but also a characteristic architecture to be well-maintained. Other details about the Baroque Library can be found in Ref. [4].

## 2 Experimental design, materials and methods

Indoor air temperature (T) and relative humidity (RH) were monitored in the Baroque Library during two periods:

• a first monitoring period (MP1), from December 17^th^, 2016 to March 14^th^, 2017;
• a second monitoring period (MP2), from April 3^rd^, 2017 to July 6^th^, 2017.

**Fig. 1:**
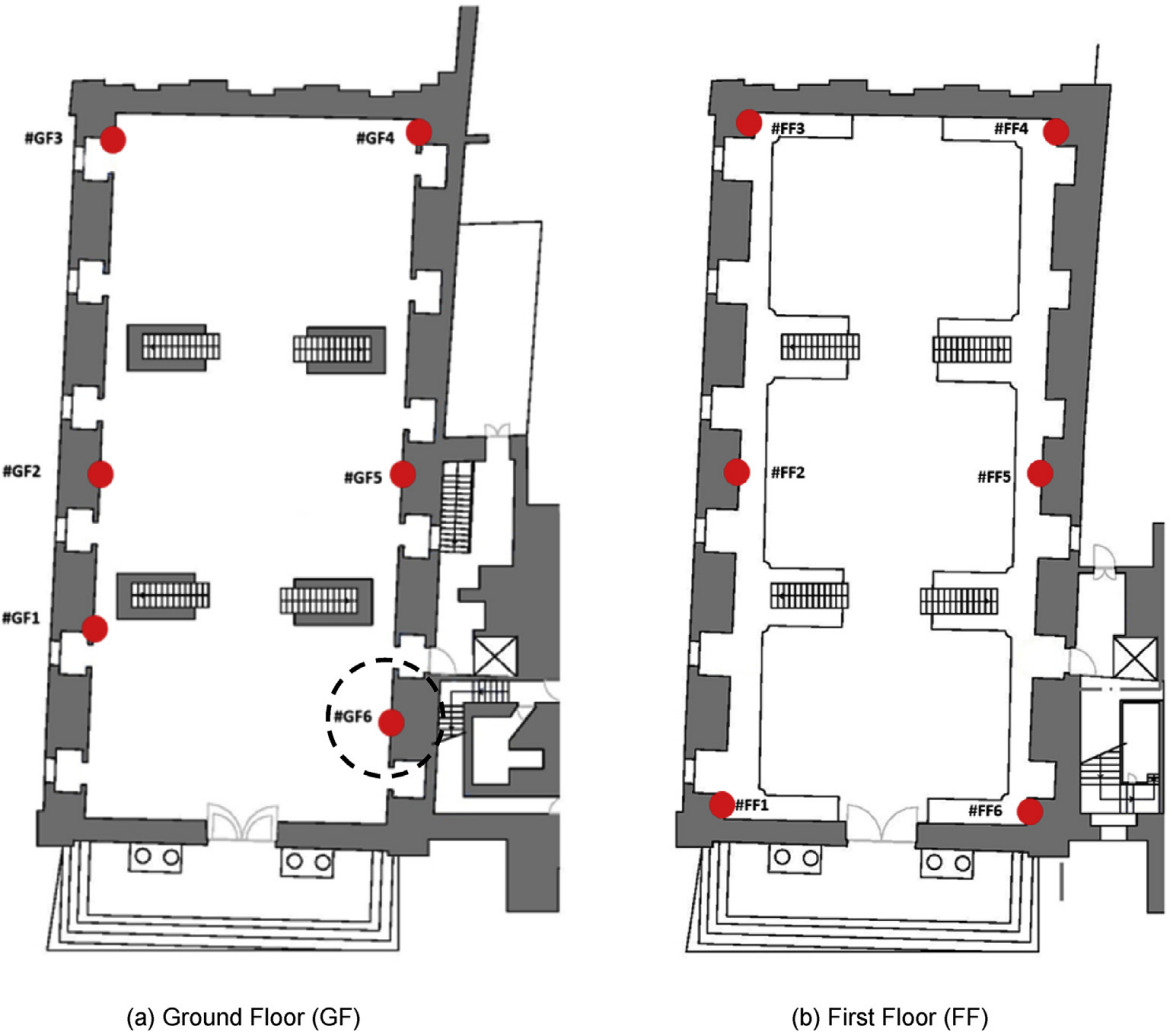
Sensors location in the Baroque Library, University of Coimbra, Portugal.

The used sensors were HOBO UX100-003 data loggers, which automatically gathered data every 5 minutes [5]. The characteristics of the sensors are reported in Table 1. The position of the sensors in the Baroque Library are shown in Fig. 1: six data-loggers were posed at ground floor (“GF”, in the following), and other six data-loggers were posed at mezzanine floor (“FF”, in the following). Sensor GF6 (highlighted with a black dotted circle in Fig. 1) did not record data due to technical problems, whereas all the other sensors properly worked during the whole monitoring campaign.

**Table 1:** Technical characteristics of the data loggers.

Sensor/Parameter	Measuring range	Accuracy
Temperature	−20° to 70 °C	±0.21 °C from 0° to 50 °C
Relative Humidity	15%–95%	±3.5% from 25% to 85% including hysteresis at 25 °C; below 25% and above 85%, ±5% typical

In this paper, the main focus is the characterisation of the microclimate profiles, both on temporal and spatial point of view.

**Fig. 2:**
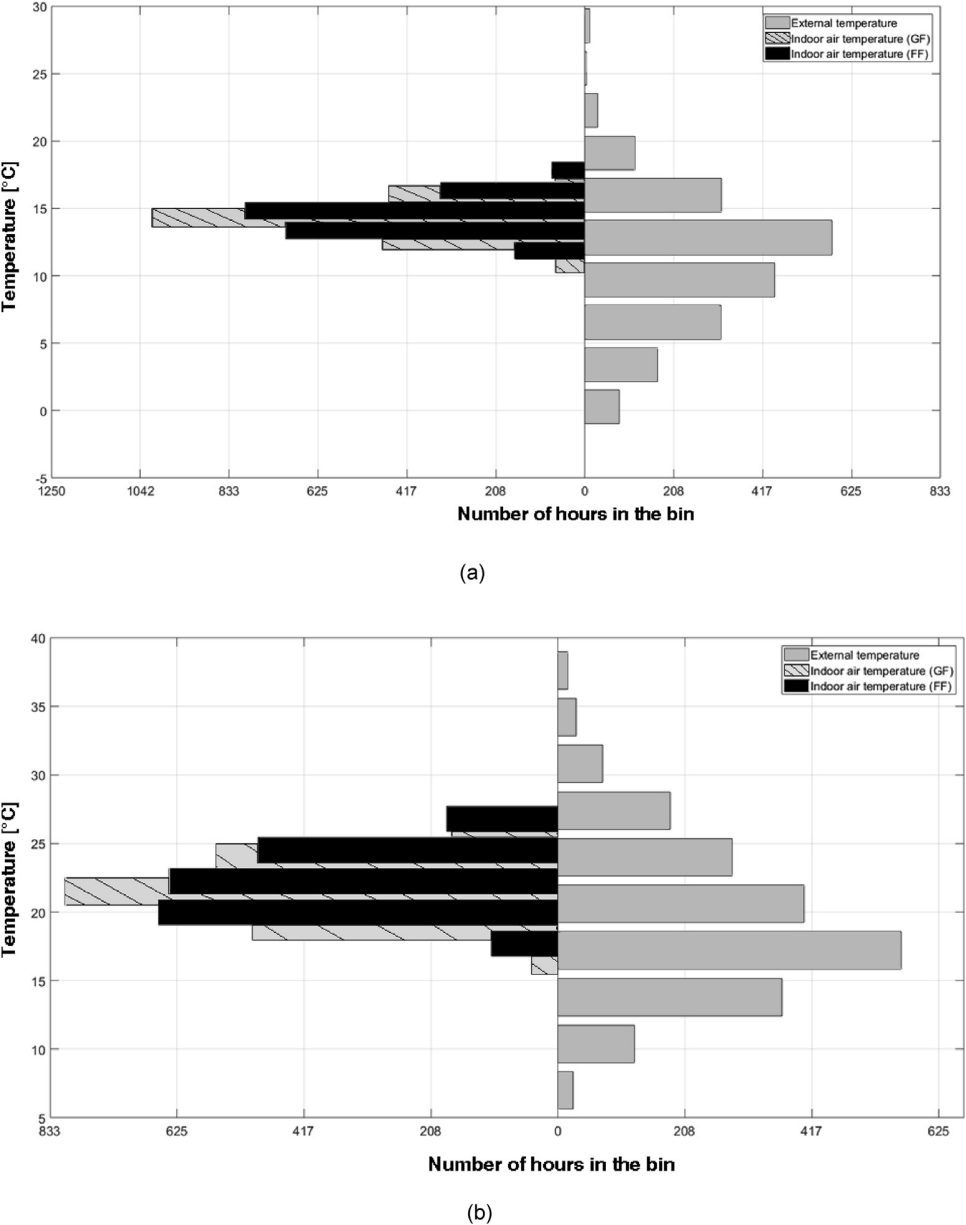
Histograms of external temperature and indoor air temperature (GF and FF): *a)* MP1; *b)* MP2.

A first analysis of the monitoring data has shown the effectiveness of the thick walls in smoothing and delaying the changes in outdoor climate. Fig 2 shows two histograms: on the right side, the bars represent the number of hours where the corresponding external air temperature bin is found, while on the left side, the bars represent the number of hours where the corresponding indoor air temperature bin is found.

As for the temporal characterisation, periodicities on the basis of the hour of the day and the day in the week were sought. To do so, three reference weeks were chosen for the MP1:

• From 17^th^ December to 24^th^ December
• From 20^th^ January to 27^th^ January
• From 1^st^ March to 8^th^ March;

Analogously, three reference weeks were chosen for the MP2:

• From 4^th^ April to 11^th^ April
• From 8^th^ May to 15^th^ May
• From 17^th^ June to 24^th^ June.

**Fig. 3:**
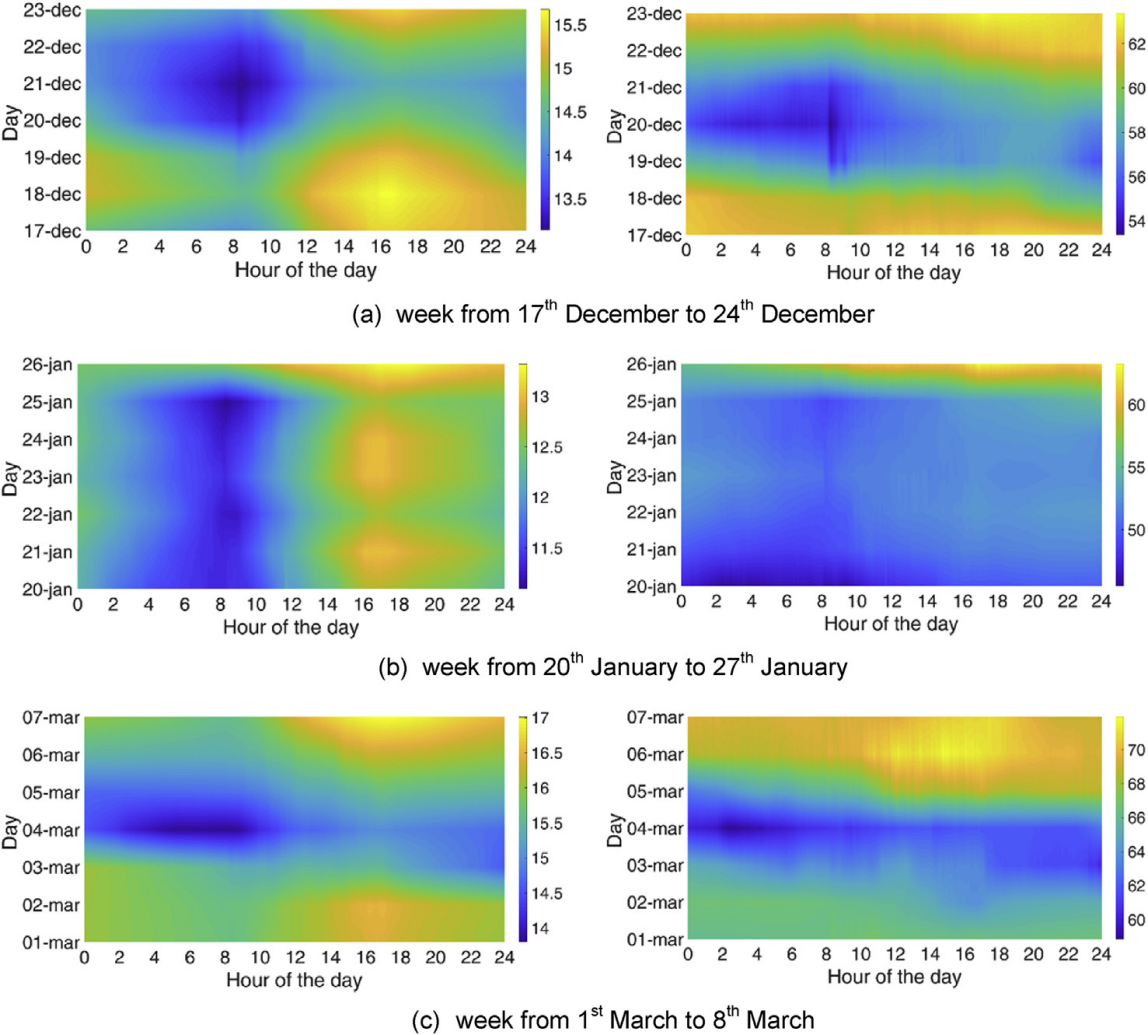
Temperature and relative humidity profiles for 3 weeks in the MP1: analysis of the daily and weekly periodicity.

**Fig. 4:**
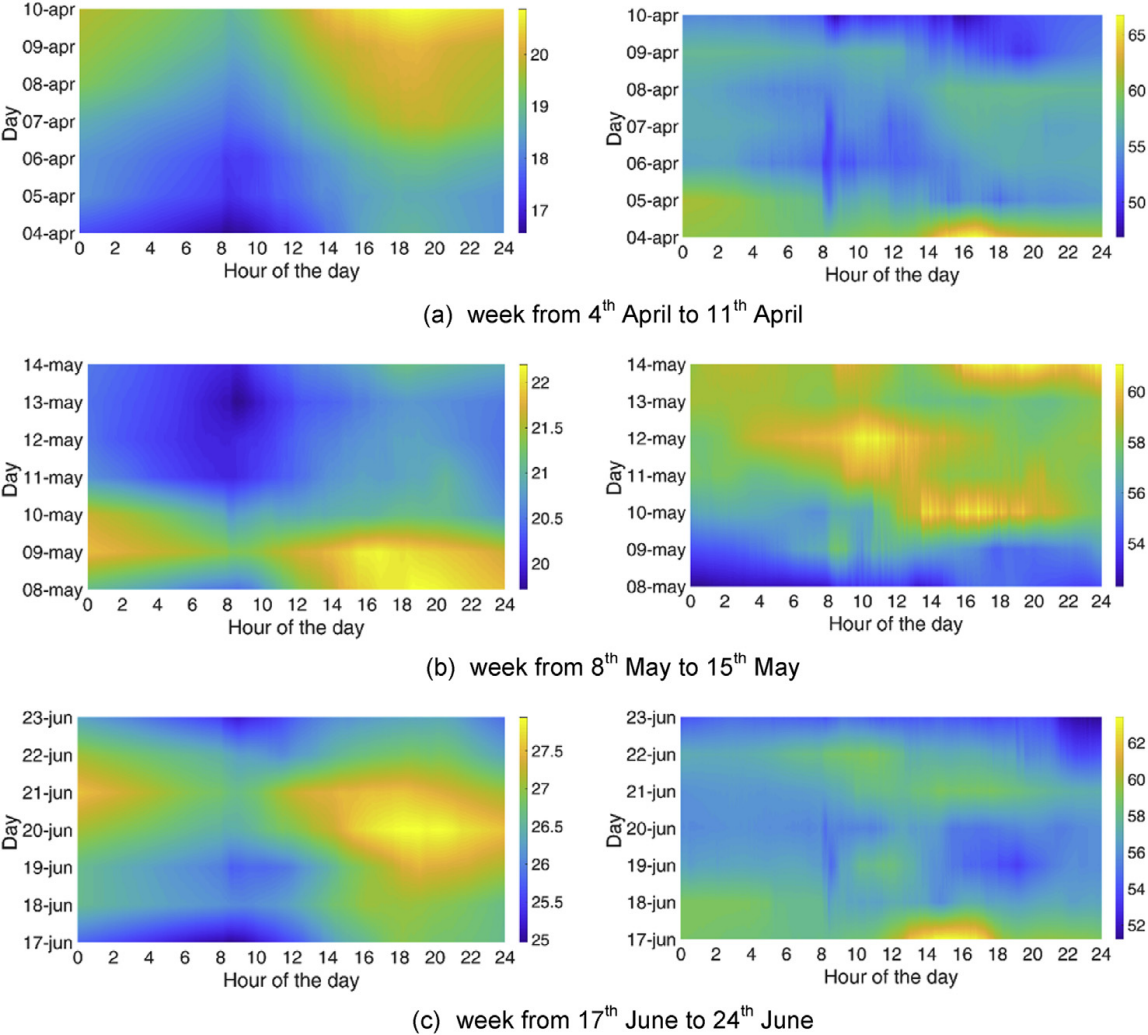
Temperature and relative humidity profiles for 3 weeks in the MP2: analysis of the daily and weekly periodicity.

Typical carpet plots were created, with the hour on the x-axis and the day on the y-axis. The colour represents the chosen variable (temperature or relative humidity). These graphs are reported in Fig. 3, Fig. 4. These images were used to identify daily and/or weekly periodicities. Except for the higher temperature normally reached in the afternoon hours of the day, these Figures did not highlight any significant periodicity.

**Fig. 5:**
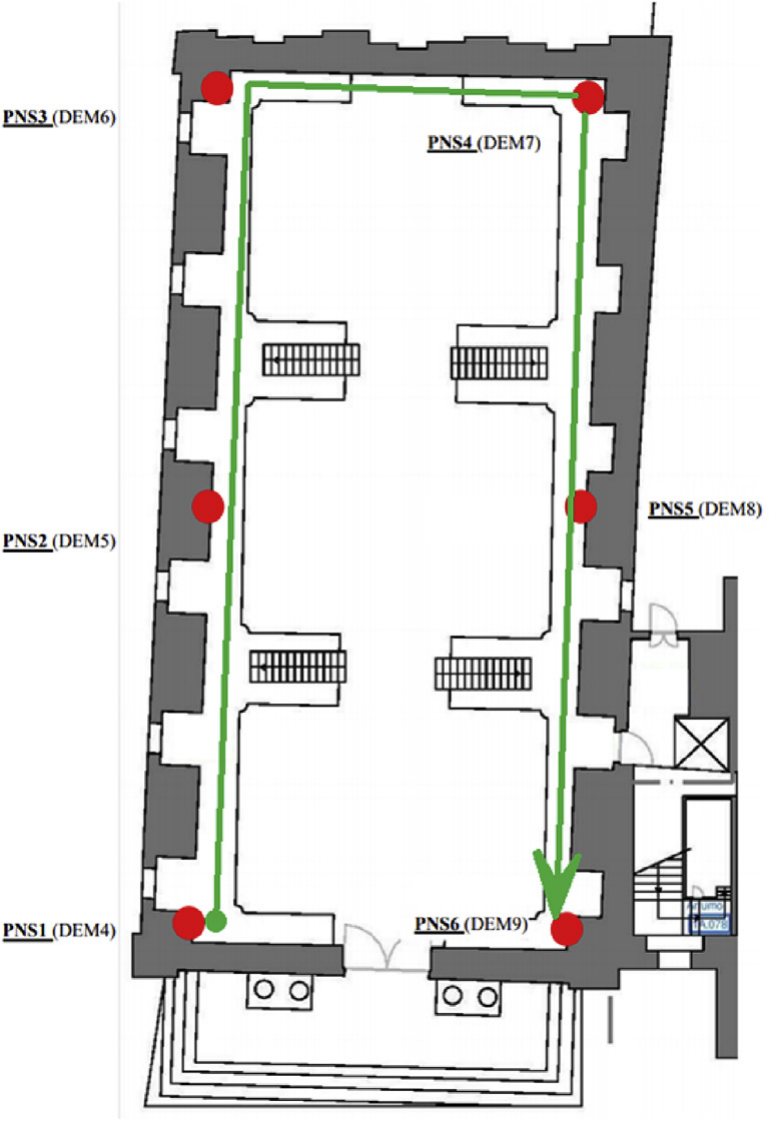
Parametrization of perimeter in the Library. The round green marker (begin arrow) identifies the point at 0 m in Fig. 5, Fig. 6, Fig. 7, Fig. 8, while the green open arrowhead identifies the point at 77 m.

**Fig. 6:**
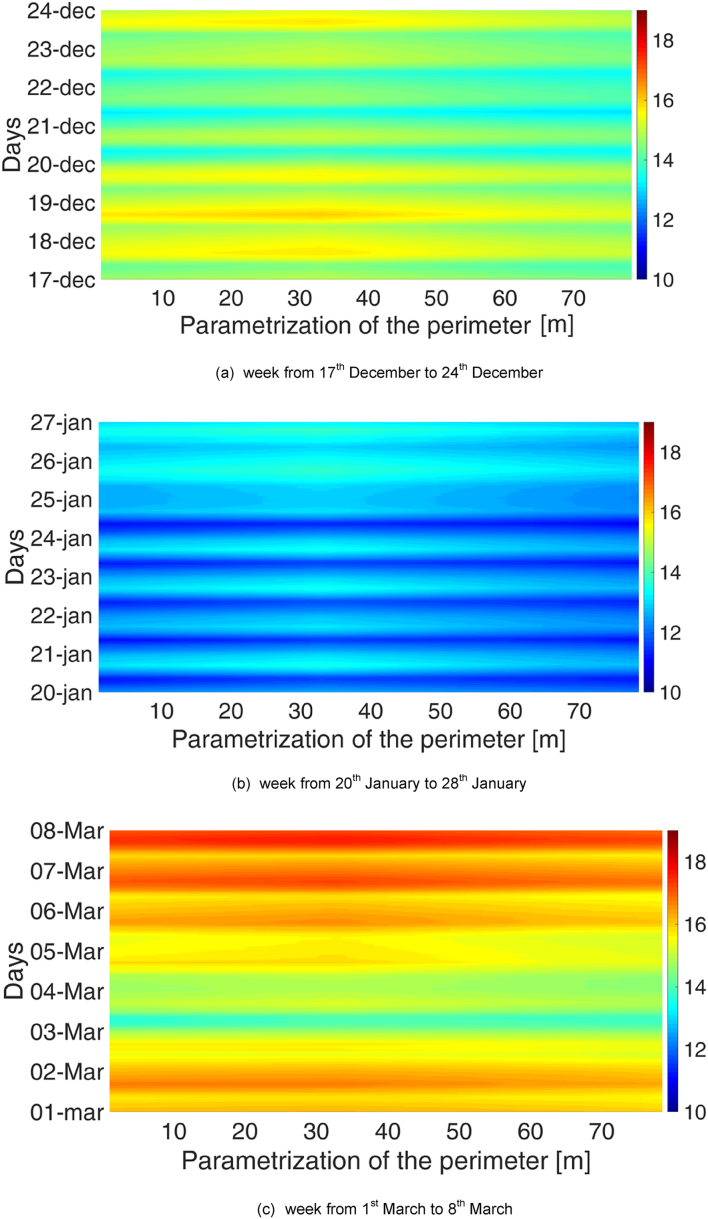
Distribution of air temperature along the perimeter for three reference weeks in the MP1.

**Fig. 7:**
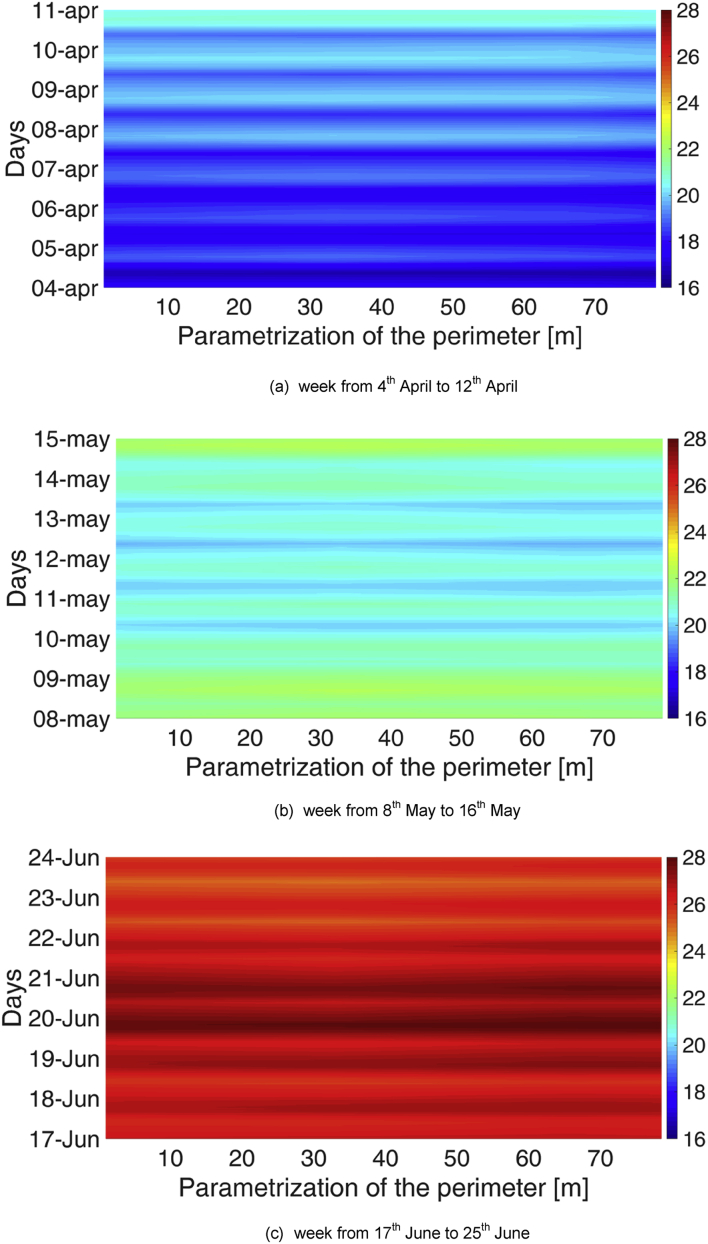
Distribution of air temperature along the perimeter for three reference weeks in the MP2.

**Fig. 8:**
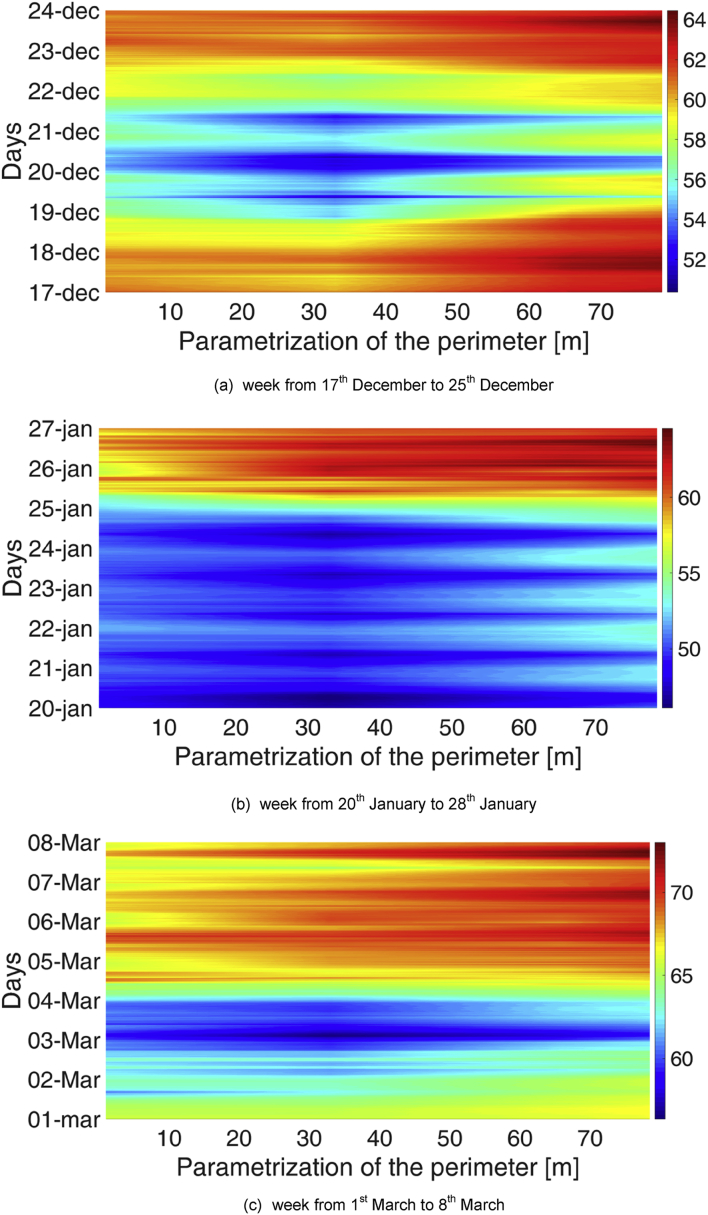
Distribution of relative humidity along the perimeter for three reference weeks in the MP1.

**Fig. 9:**
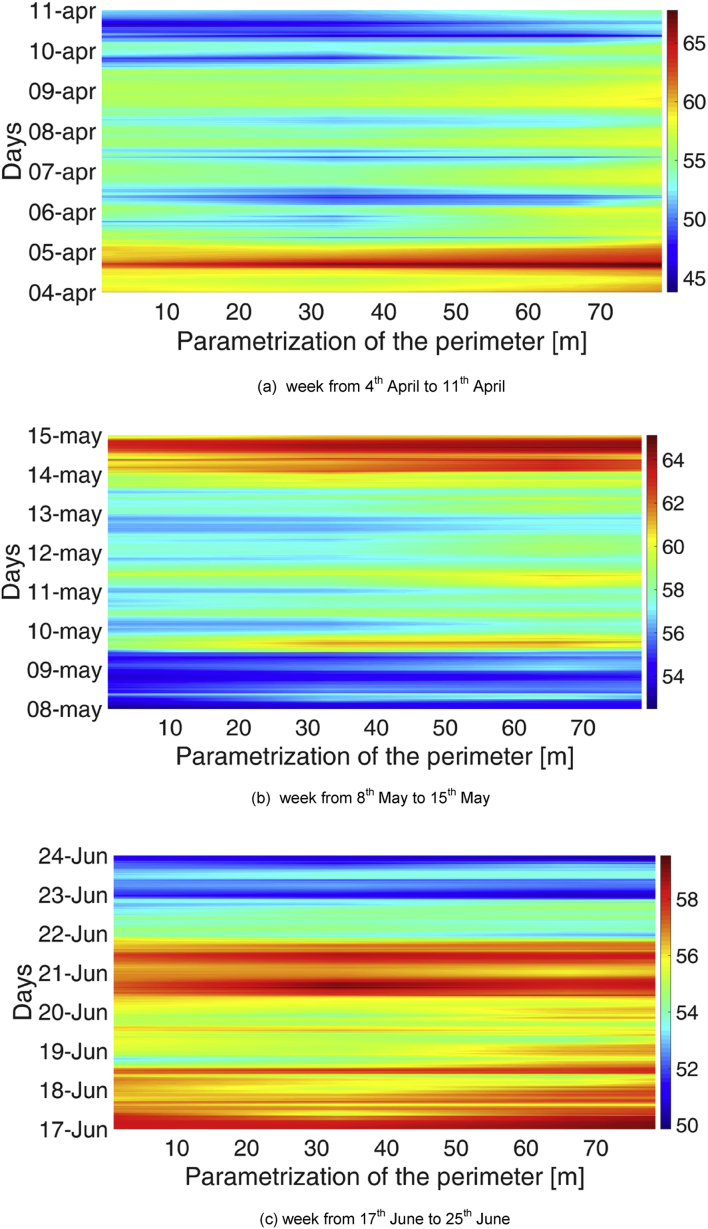
Distribution of relative humidity along the perimeter for three reference weeks in the MP2.

Spatial gradients were monitored along the perimeter of the Library. Also in this case, carpet plots were used, with a parametrization of the library perimeter according to Fig. 5. The spatial distribution of temperature at a specific point on the x-axis is reported in Fig. 6, Fig. 7, respectively for the MP1 and MP2. Analogously, Fig. 8, Fig. 9 show the spatial distribution of relative humidity at a specific point on the x-axis for the MP1 and MP2, respectively.

**Fig. 10:**
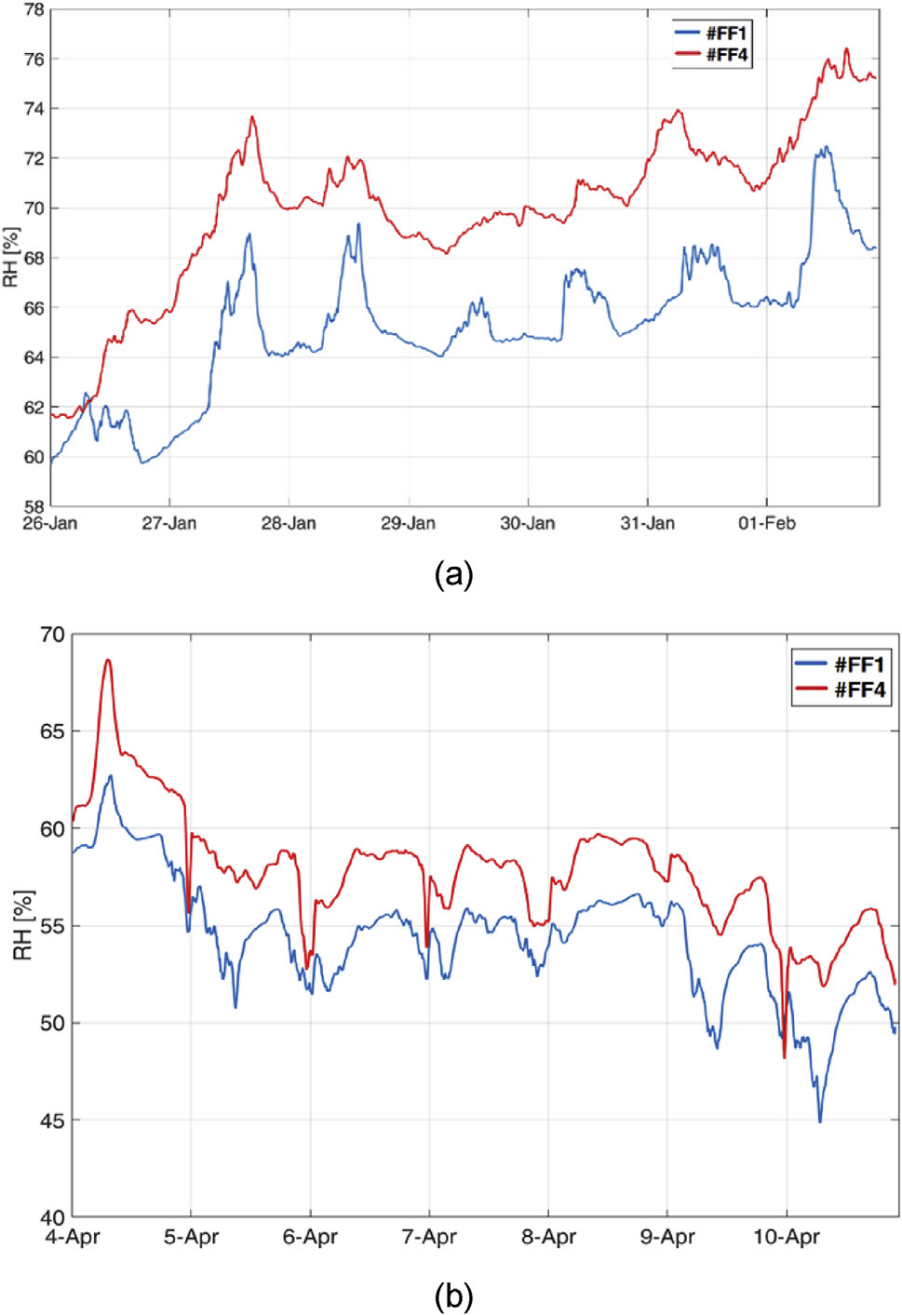
RH profiles at the opposite walls of the Library, for two reference weeks: (a) for MP1, (b) for MP2.

Higher values of both temperature and relative humidity are reached between 25 and 45 m on the perimeter, corresponding to the front wall opposite the opening door, where the influence of external climate is minimum. These values are probably due to the lower influence of ventilation through the main door openings and closings. The difference between RH profile between the entrance (data-loggers #FF1 and #FF 6, data-logger #GF1) and the opposite wall (data-loggers #FF3 and #FF4, data-loggers #GF3 and #GF4) is clearly visible also in Fig. 10, where two examples of RH profiles are reported, one for MP1 and one for MP2.

In some cases, slightly higher temperatures are found during weekends (17^th^ −18^th^ December, 14^th^ May).

**Fig. 11:**
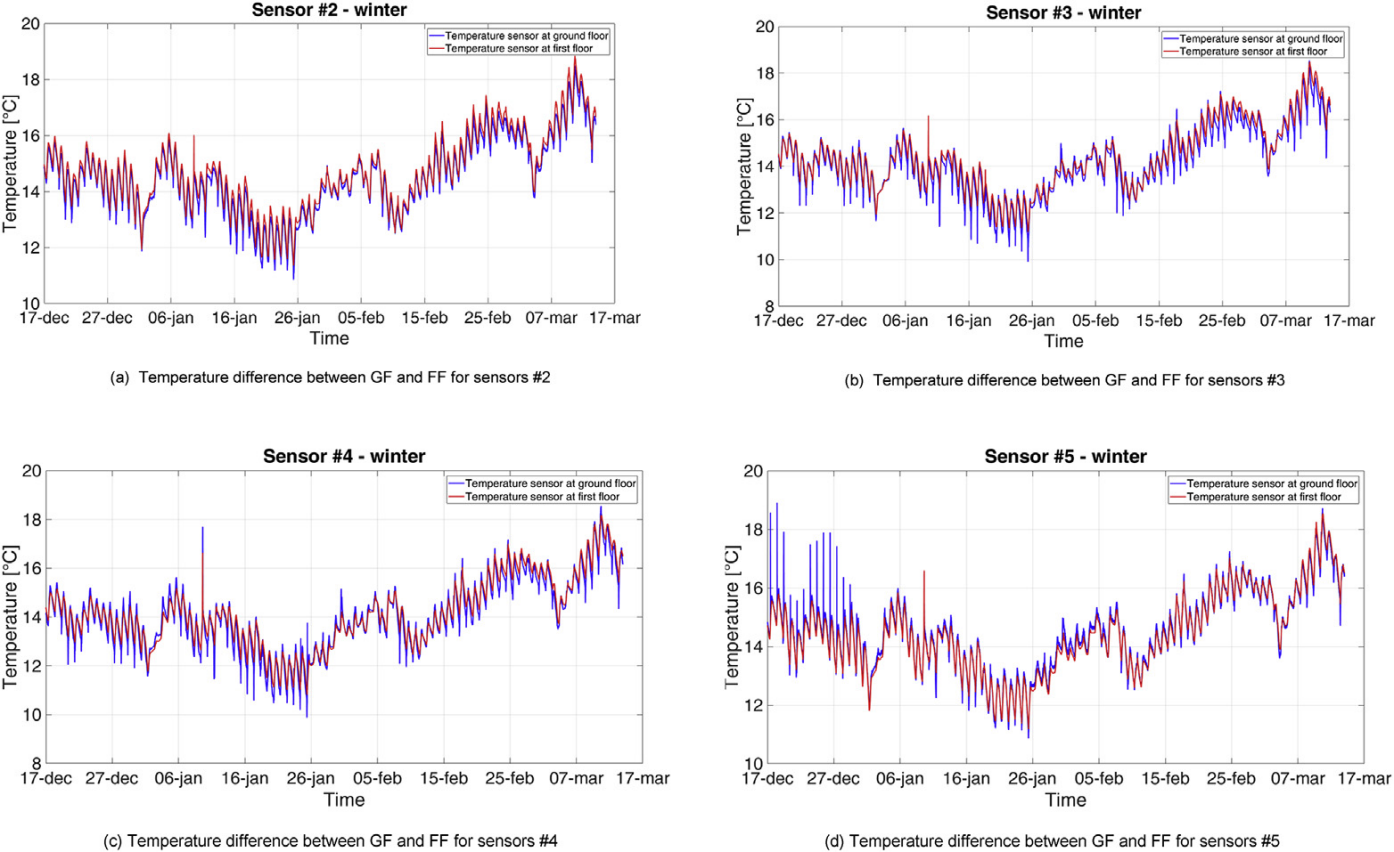
Profiles of temperature at ground and first floor during the MP1.

**Fig. 12:**
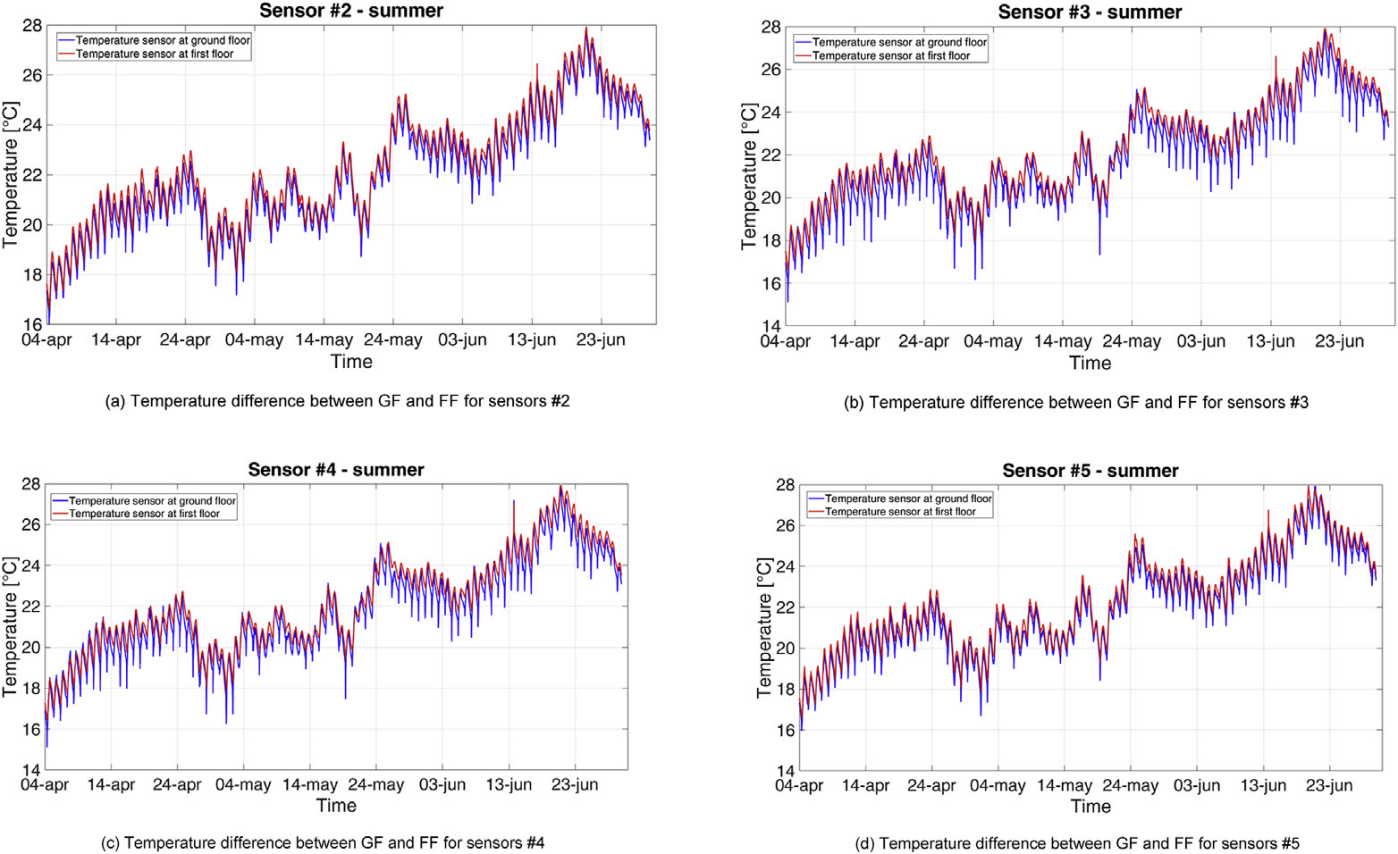
Profiles of temperature at ground and first floor during the MP2.

Complementary, another analysis was carried out, to verify the presence of stratification between the temperature at ground and first floor. The results showed that this phenomenon was almost absent during the MP1, while it is more pronounced in the MP2, due to the higher indoor temperatures. Results are shown in Fig. 11, Fig. 12. From December 16^th^ to December 30^th^, sensor #5 measured a temperature peak every day at 11 a.m. (see Fig. 11d): this peak was about 1.5–2 °C higher than the previous time step and represents an anomaly, as the other sensors did not measure so high temperatures. This event could have been justified by direct incidence of sunlight (the sensor was located in a south-oriented shelf).
