# Supplementary material for: Data of temperature and relative humidity in a historic library in Portugal
Source: Data Brief. 2019 Mar 15;24:103788. doi: 10.1016/j.dib.2019.103788 (PMC6446128; doi:10.1016/j.dib.2019.103788)
Supplement: Multimedia component 1 [file mmc1.pdf]

Conflict of Interest and Authorship Declaration Form

On behalf of all the co-authors, I confirm that there are no known conflicts of interest associated with this publication and there has been no significant financial support for this work that could have influenced its outcome.

I confirm that the manuscript has been read and approved by all named authors and that there are no other persons who satisfied the criteria for authorship but are not listed. I further confirm that the order of authors listed in the manuscript has been approved by all the authors.

All authors have participated in (a) conception, analysis and interpretation of the data; (b) drafting the article or revising it critically for important intellectual content; and (c) approval of the final version.

Eva Schito

Eva Schito
